# Calycosin alleviates titanium particle‐induced osteolysis by modulating macrophage polarization and subsequent osteogenic differentiation

**DOI:** 10.1111/jcmm.18157

**Published:** 2024-03-17

**Authors:** Hui Jiang, Yang Wang, Zhao Tang, Xianjiang Peng, Chan Li, Yangjie Dang, Rui Ma

**Affiliations:** ^1^ Department of Orthopedics The Affiliated Jinling Hospital of Nanjing Medical University Nanjing China; ^2^ Department of Anesthesiology, Xi'an Children's Hospital Affiliated Children's Hospital of Xi'an Jiaotong University Xi'an China

**Keywords:** Calycosin, Macrophage, Osteogenesis, Osteolysis, Ti

## Abstract

Periprosthetic osteolysis (PPO) caused by wear particles is one of the leading causes of implant failure after arthroplasty. Macrophage polarization imbalance and subsequent osteogenic inhibition play a crucial role in PPO. Calycosin (CA) is a compound with anti‐inflammatory and osteoprotective properties. This study aimed to evaluate the effects of CA on titanium (Ti) particle‐induced osteolysis, Ti particle‐induced macrophage polarization and subsequent osteogenic deficits, and explore the associated signalling pathways in a Ti particle‐stimulated calvarial osteolysis mouse model using micro‐CT, ELISA, qRT‐PCR, immunofluorescence and western blot techniques. The results showed that CA alleviated inflammation, osteogenic inhibition and osteolysis in the Ti particle‐induced calvarial osteolysis mouse model in vivo. In vitro experiments showed that CA suppressed Ti‐induced M1 macrophage polarization, promoted M2 macrophage polarization and ultimately enhanced osteogenic differentiation of MC3T3‐E1 cells. In addition, CA alleviated osteogenic deficits by regulating macrophage polarization homeostasis via the NF‐κB signalling pathway both in vivo and in vitro. All these findings suggest that CA may prove to be an effective therapeutic agent for wear particle‐induced osteolysis.

## 1 INTRODUCTION

Total joint arthroplasty is a successful treatment for end‐stage arthritis.^1^ However, with the increasing number of arthroplasties and the passage of time, more patients require revision arthroplasty due to the aseptic loosening of the prosthesis.^2^ The materials used for joint replacement implants include titanium (Ti) alloy, ultra‐high polyethylene polymer and bone cement. These implants are subject to wear and corrosion, resulting in wear debris. Osteolysis caused by wear debris around the prosthesis is the leading cause of the aseptic loosening of the prosthesis.^3^ Considering the inevitability of wear debris, reducing the impact of wear debris may be a good way to extend the lifespan of prostheses.

Wear debris interacts with various cell types in the microenvironment, including macrophages, osteoclasts, osteoblasts and fibroblasts, which may ultimately disrupt the balance between bone resorption and bone formation.^4^ Indeed, wear debris may stimulate M1/M2 macrophage polarization imbalance, leading to osteogenic deficiency, which plays a crucial role in developing aseptic loosening. M1 macrophages act as a paracrine inhibitor of osteogenesis,^5^ while M2 macrophages augment tissue repair and promote osteogenesis by secreting anti‐inflammatory cytokines.^6^ Regulating the polarization balance of macrophages may serve as an effective strategy to alleviate wear particle‐induced osteolysis and promote bone formation.^7^

Natural products are a main source of new drug development, and natural small molecules derived from traditional Chinese medicines have a wide spectrum of biological activities and exhibit promising prospects in disease prevention and treatment.^8^ *Astragalus membranaceous* (AM) is a common food‐origin Chinese medicine with a long history of treating osteoporosis, inflammation and other related diseases.^9^ Calycosin (CA) is the main active compound of AM, a plant oestrogen with various pharmacological properties such as anti‐inflammatory, anti‐osteoporotic and antioxidant activities,^10^ implying that it may have an inhibitory effect on inflammatory‐related osteolysis diseases such as PPO. But to the best of our knowledge, the therapeutic effect of CA on PPO, especially its role in Ti particle‐induced macrophage polarization and subsequent osteogenesis promotion, remains elusive.

This study aimed to investigate the effect of CA on PPO through a Ti‐particle‐induced calvarial osteolysis mouse model in vivo, and clarify the impact of CA on wear particle‐induced macrophage polarization imbalance and subsequent osteogenic deficit. The results showed that CA alleviated the inflammatory response and attenuated osteolysis in vivo. At the same time, CA modulated Ti‐induced macrophage polarization and subsequently alleviated the inhibition of osteogenic differentiation in Ti‐induced macrophage‐conditioned medium in vitro. Mechanistically, the regulatory effect of CA on Ti particle‐induced macrophage polarization may be achieved by inhibiting the NF‐κB signalling pathway. The findings may provide useful clues for the development of therapeutic agents for PPO.

## 2 MATERIALS AND METHODS

### 2.1 Chemicals and reagents

CA (purity >98%) was purchased from Aladdin Biotech (China). A stock solution of CA at a concentration of 0.1 M was prepared using dimethyl sulfoxide (DMSO), which was further diluted with a cell culture medium for cell experiments and a sterile phosphate‐buffered solution (PBS) for animal experiments. Ti particles (purity 99.99%, Aladdin Biotech, China) were scanned by scanning electron microscopy (SEM) to determine their diameters. To eliminate endotoxin from Ti particles, they were baked at 200°C for 10 h, submerged in 75% ethanol for 48 h, washed with sterile water four times and then suspended in sterile PBS at 30 mg/mL.^11^ Before use, the Ti suspension was sonicated in an ultrasound bath for 10 min.

### 2.2 Cell lines and culture

RAW264.7 macrophage and MC3T3‐E1 cell lines were acquired from the Cell Bank of the Chinese Academy of Sciences (Shanghai, China). Macrophages were cultured in Dulbecco's Modified Eagle Medium (DMEM; HyClone; USA) supplemented with 10% fetal bovine serum (FBS; Gibco; USA) and 1% penicillin/streptomycin (HyClone; USA). MC3T3‐E1 cells were cultured in alpha‐Minimum Essential Medium (α‐MEM; Hyclone; USA) with 10% FBS. To induce osteogenic differentiation, the medium was added with 10 nmol/L dexamethasone (D4902; Sigma), 10 mmol/L β‐glycerophosphate (G9422; Sigma) and 50 μg/mL ascorbic acid (A4034; Sigma), and refreshed every other day.

### 2.3 Cell viability assays

RAW264.7 cells were seeded into 96‐well plates at a density of 4000/well and cultured with indicated reagents for a specific time. At the end of the experiment, cell viability was determined using a CCK‐8 (C0038; Beyotime, China) kit according to the manufacturer's instructions.

### 2.4 Osteogenic differentiation of MC3T3‐E1 cells in conditioned medium

RAW264.7 cells were cultured with Ti and different concentrations of CA for 24 h, and then replaced with a new α‐MEM medium containing 10% FBS for 48 h. The supernatant was collected, centrifuged at 2000 rpm for 20 min at 4°C and filtered through a 0.22 μM filter. The ordered medium was mixed with an osteogenic medium in 1:1 to create a conditioned medium (CM). The MC3T3‐E1 cell suspension was seeded into 24‐well plates with 1 × 10^5^ cells per well and cultured with CM. The medium was altered every 48 h.

### 2.5 Alkaline phosphatase (ALP) staining and activity assay

MC3T3‐E1 cells were seeded in 24‐well plates and cultured with osteogenic medium or CM for 7 days, after which the medium was discarded. The cells were added with 4% paraformaldehyde at room temperature for 10 min, then permeabilized with 0.1% Triton‐X‐100 for 6 min and incubated with PBST for another 10 min. Finally, 300 μL BCIP/NBT (C3206, Beyotime, China) alkaline phosphatase staining solution was added to each well and incubated at 37°C for 40 min. Photographs were taken after removing the stain solution. The ALP activity was measured with an ALP assay kit (A059‐2‐2; Nanjing Jiancheng Bioengineering Ltd, Nanjing, China) according to the manufacturer's protocol.

### 2.6 Lentivirus knockdown

RAW264.7 cells were seeded into 6‐well plates at a density of 3 × 10^5^ cells per well; lentiviral vectors (TransSheep Biotech, Shanghai, China) containing 100 nM NF‐κB p65 short hairpin RNA (shRNA, 5′‐ACACTGCCGAGCTCAAGATCT‐3′) were used to transfected cells using the Lipofectamine® 3000 reagent (Invitrogen, Thermo Fisher Scientific). After 24 h, the medium was changed into a typical medium. The knockdown of P65 was analysed using western blot and qRT‐PCR.

### 2.7 Ti particle‐induced calvarial osteolysis model in mice

The animals used in this study were 8‐week‐old male C57/BL6 mice (21–23 g) housed in a specific pathogen‐free (SPF) animal facility at the Laboratory Animal Center of Jinling Hospital. The mice were fed commercially available pellet food and clean water. The experiment protocol was approved by the Experimental Animal Ethics Committee of the said hospital. The mouse calvarial osteolysis model was established as previously reported.^12^ The 24 mice were equally randomized into four groups (*n* = 6 per group): (1) Control group, (2) Ti‐treatment group, and (3 and 4) Ti and CA (12.5 mg/kg)/CA (50 mg/kg) co‐treatment group. After anaesthesia isoflurane, a 1 cm incision was made on the scalp of the animal. Control mice were sutured directly on the scalp, while Ti and CA mice were sutured after injection with PBS suspension containing 30 mg Ti particles into the calvarias. Mice in the CA group underwent intraperitoneal injections of CA (12.5 or 50 mg/kg/day) from day 2 to day 14. In addition, mice in the Control and Ti‐treatment groups received an equal volume of vehicles. After 2 weeks, all mice were killed, from which the calvarias and serum were collected. The dose of CA used in the animal experiment was determined based on previous reports.^13^, ^14^

### 2.8 Micro‐CT

After the collection of the calvarias, the soft tissue was isolated, and the samples were scanned using a high‐resolution micro‐CT (Sky Scan, Belgium), with the X‐ray energy set at 50 kV and 500 mA, and the scanning parameters at a resolution of 9 mm. Three‐dimensional (3D) images were reconstructed using the manufacturer's software. Following reconstruction, a square region of interest (ROI, 3 × 3 × 1 mm) around the midline suture was chosen for further quantitative analysis as previously reported.^15^ Bone mineral density (BMD), bone volume/tissue volume (BV/TV) and trabecular thickness (Tb. Th) were determined using the manufacturer's software. The number of pores and pore area within the ROI were counted using the ImageJ software as previously reported.^3^

### 2.9 Histopathological evaluation

Bone formation was evaluated dynamically using calcein double‐label staining. Calcein (Sigma‐Aldrich) was intraperitoneally injected at 10 mg/kg on days 1 and 12 after surgery. Images of calcein double labelling were observed under a fluorescence microscope, The Bioquant Osteo software was utilized to calculate the mineral apposition rate (MAR). Specifically, MAR is calculated as the fluorescence staining distance between the two calcium divided by the interval of labelling time as previously reported.^16^ The collected calvarias were decalcified for 2 weeks in 10% EDTA before being paraffin‐embedded and cut into 5–μm‐slices. The tissue was stained by haematoxylin and eosin (H&E) and Masson's (Alliance Biotech) stain according to the manufacturer's instructions. The images were captured using a high‐quality Olympus microscope (Carl Zeiss, Germany). The inflammation area, bone erosion area and collagen volume fractions were calculated by two competent experiments blind to treatment, they jointly used ImageJ software to circle the area of inflammation, erosion or collagen area, and then the inflammation area, bone erosion area in haematoxylin and eosin‐stained calvarias and the collagen volume fraction (%) in Masson‐stained calvarias were calculated.

### 2.10 Immunofluorescence staining

Immunofluorescence was performed using the standard protocol as previously reported.^17^ RAW264.7 cells were seeded at a density of 1 × 10^4^ cells per well in a 24‐well plate. Following a 2‐h treatment with Ti particle and specified reagents, the cells were fixed with 4% paraformaldehyde for 10 min, permeabilized with Triton‐X100 for 10 min and treated with bovine serum albumin for 30 min. The appropriate primary antibody was added and incubated for 6 h, followed by appropriate secondary antibodies for 2 h. DAPI was added for 5 min to visualize the nuclei. Fluorescence intensity was quantified using ImageJ (Bethesda, MD) after capturing images with a fluorescence microscope (DP71; Olympus, Tokyo, Japan).

### 2.11 ELISA

At the end of the experiment, blood was obtained from the mice and their serum was collected following centrifugation at 3000 rpm for 30 min. The ELISA kits for measuring IL‐1β (E‐EL‐M0037c), IL‐6 (E‐EL‐M0044c), TNF‐α (E‐EL‐M3063), IL‐10 (E‐EL‐M0046c) and IL‐4 (E‐EL‐M0043c) were purchased from Elabscience (Wuhan, China). The levels of IL‐1β, IL‐6, TNF‐α, IL‐10 and IL‐4 in the mouse serum were quantified using the respective ELISA kits according to the manufacturer's instructions. The absorbance was determined on a microplate reader at OD450 nm.

### 2.12 Real‐time quantitative PCR

Total RNA was isolated from cells or tissues with Trizol (Invitrogen), the cells were harvested and the calvaria tissues were cut according to the ROI region (50 mg), then digested in 1 mL Trizol reagent (Invitrogen) and total RNA was extracted according to the manufacturer's instructions. The absorbance ratio (260/280) was measured to verify the purity of RNA samples. Then, RNA was reverse transcribed to cDNA using the PrimeScript™RT Master Mix kit (Takara). For relative quantitative real‐time PCR (qRT‐PCR), the total volume (20 μL) of each PCR reaction comprised of 10 μL SYBR Green qPCR Mix, 8 μL ddH2O, 1 μL cDNA and 0.5 μL of each primer following the manufacturer's protocol, as previously described.^18^ The relative mRNA levels were qualified using the 2^−ΔΔCt^ method. The primer sequences utilized are listed in the Supplementary Table S1.

### 2.13 Western blot

The Western blot analysis was performed according to a previous report.^19^ The proteins were extracted from cells or tissues, and their concentrations were determined using a BCA protein quantification kit (Yeasen, China). An equal amount of protein (50 μg) was adjusted on SDS‐PAGE and then transferred to the PVDF membrane. The membrane was blocked, and incubated with antibodies against IKKα (ab32041, Abcam), P‐IKKα/β (ab194528, Abcam), P65 (ab16502, Abcam), P‐P65 (ab76302, Abcam), IκBα (ab32518, Abcam), IκBα (ab133462, Abcam) or GAPDH (ab128915, Abcam) overnight at 4°C, washed and probed with secondary antibodies. The ECL system was used to scan the membranes and capture images. The intensity of each band was calculated as a fold change relative to the control.

### 2.14 Statistical analysis

We utilized GraphPad Prism 8.01 software (GraphPad Software, Inc., USA) for data analysis and plotting. Results are presented as the mean ± standard deviation (SD). Differences between the two groups were evaluated using the Student's *t*‐test, and differences between multiple groups were compared using a one‐way ANOVA. The statistical significance was achieved at *p < 0.05.

## 3 RESULTS

### 3.1 CA attenuates Ti particle‐induced calvarial osteolysis in a mouse model

**FIGURE 1:**
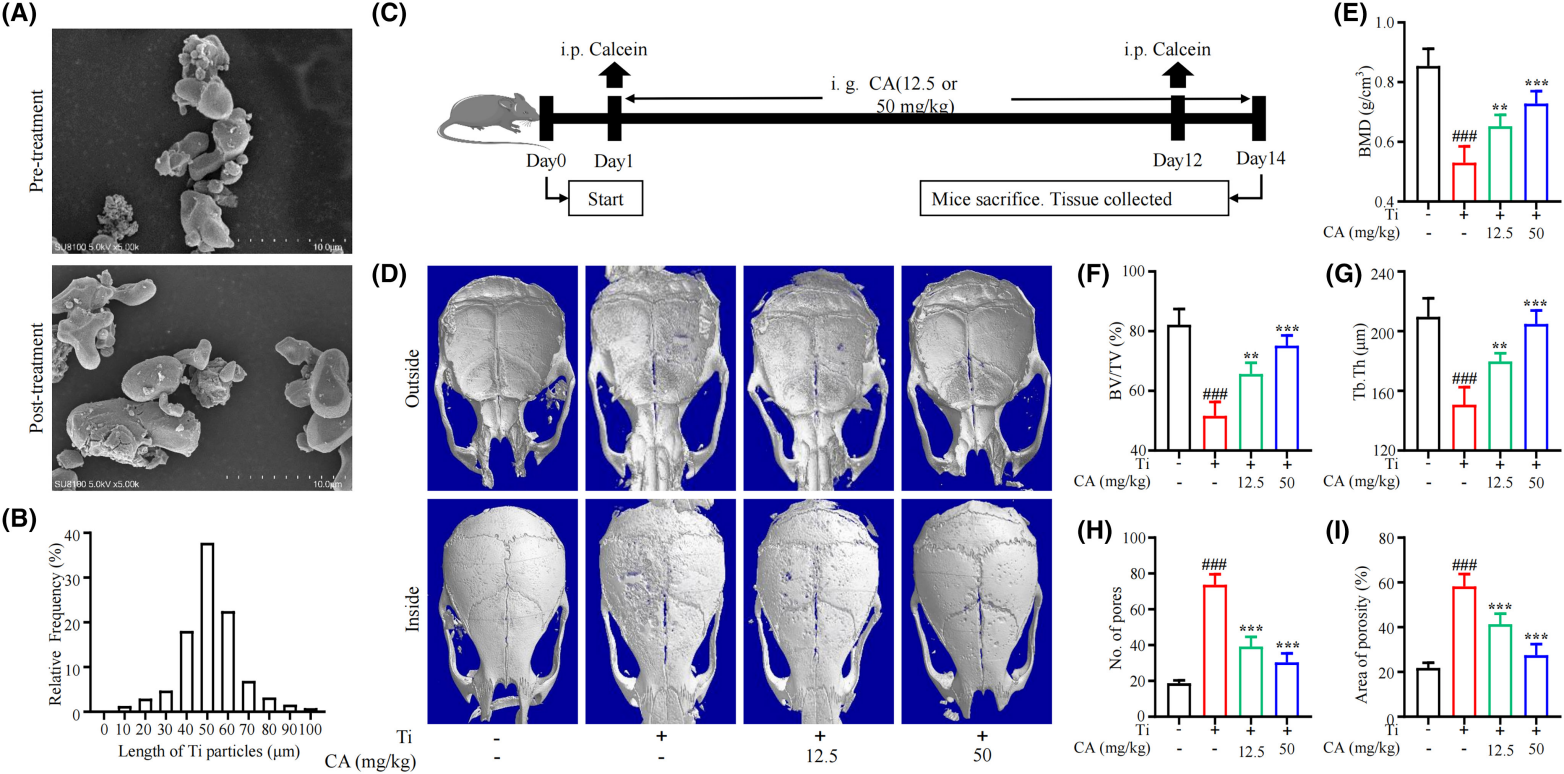
Effect of CA on Ti‐particle‐induced osteolysis in murine calvarias tissue. (A) SEM image of Ti particles. (B) Distribution of the Ti particle size. (C) Schematic depiction of the experimental schedule. (D) Representative micro‐CT 3D reconstruction images of calvarias in each group. Quantitative analysis of BMD (g/cm^2^) (E), BV/TV (%) (F), Tb.Th (μm) (G), No. of pores (H) and area of porosity (%) (I). *n* = 6 per group. *p < 0.05, **p < 0.01 versus Ctrl group. ^#^ *p* < 0.05, ^##^ *p* < 0.01 versus Ti group.

The morphology of Ti particles was observed by SEM. The results showed that Ti particles presented an irregular morphology (Figure 1A). Approximately, 80% of the particles exhibited a diameter ranging from 30 to 90 nm (Figure 1B). A Ti particle‐induced calvarial osteolysis mouse model was used to examine the therapeutic effects of CA on PPO (Figure 1C). Analysis of the mouse calvarial tissue obtained from 3D reconstruction based on micro‐CT demonstrated that CA treatment alleviated bone erosion in the middle part of the skull in the mouse model in a dose‐dependent manner (Figure 1D). Quantitative analysis showed that BMD (Figure 1E), BV/TV (Figure 1F) and Tb.Th (Figure 1G) in the ROI around the middle calvarias were significantly decreased in the Ti‐treatment group. Additionally, the number of pores (Figure 1H) and the porosity area were increased in the Ti‐treatment group (Figure 1I). Conversely, CA treatment dose‐dependently mitigated downregulation of BMD (Figure 1E), BV/TV (Figure 1F) and Tb.Th (Figure 1G) in the ROI. Correspondingly, CA treatment also reduced the number of pores (Figure 1H) and the area of porosity (Figure 1I).

### 3.2 CA attenuates Ti particle‐induced inflammation in vivo

**FIGURE 2:**
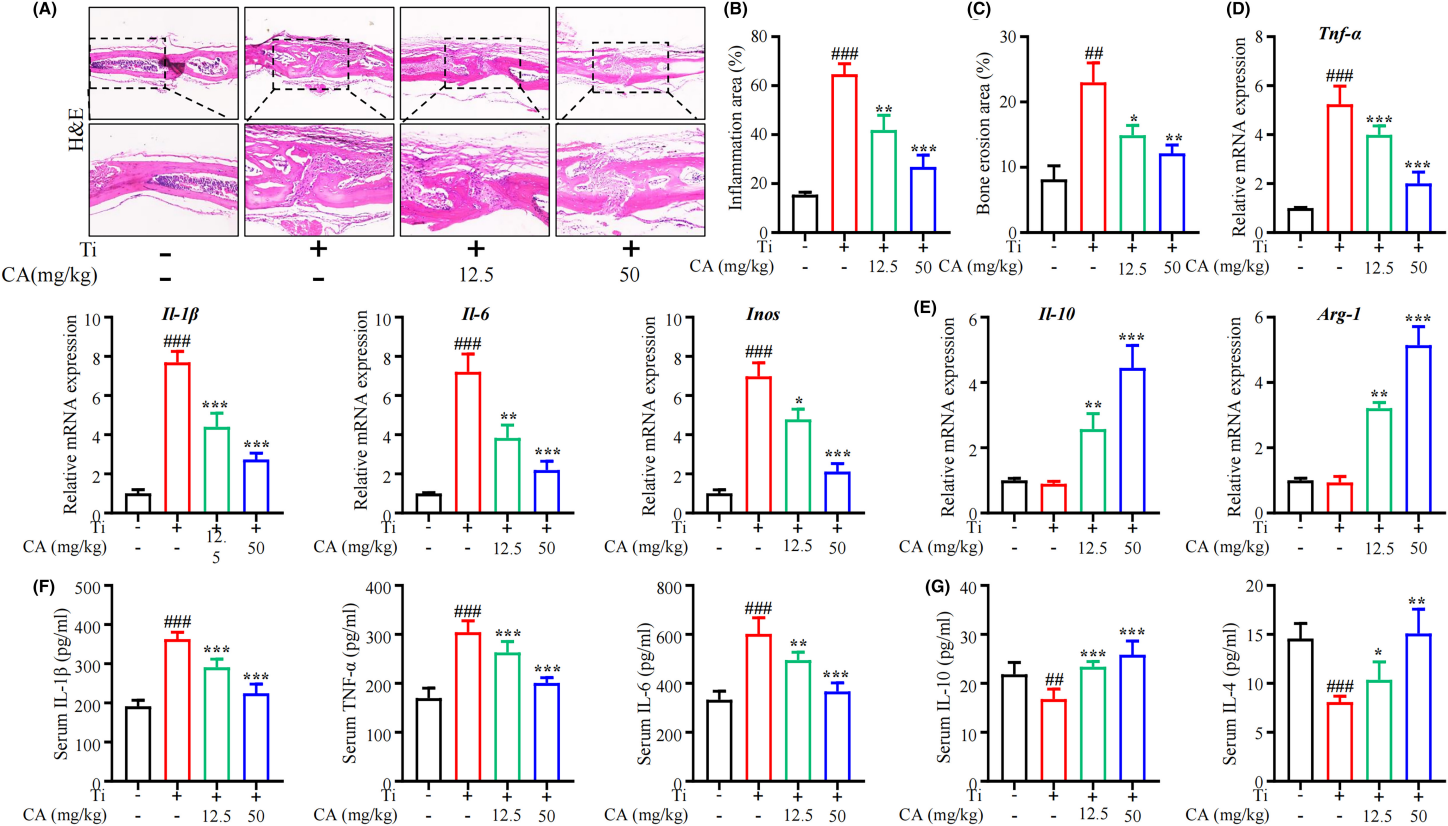
Effect of CA on Ti‐particle‐induced inflammation in murine calvarial tissue. (A) Representative images of H&E staining of calvaria tissue. (B‐C) Quantitative analysis of inflammation area (%) and bone erosion area (%). *n* = 6 per group. (D‐E) mRNA level of *Tnf‐α*, *Il‐1β*, *Il‐6*, *Inos*, *Il‐10* and *Arg‐1* in mouse calvaria tissue; *n* = 3 per group. (F‐G) Serum level of IL‐1β, TNF‐α, IL‐6, IL‐10 and IL‐4 in mice. *n* = 6 per group. ^#^ *p* < 0.05, ^##^ *p* < 0.01 versus Ctrl group. *p < 0.05, **p < 0.01 versus Ti group.

To investigate the potential mechanisms of CA in treating PPO, haematoxylin and eosin staining analysis was performed, and the result showed that CA attenuated the Ti‐induced increased inflammation area (Figure 2A,B) and bone erosion area (Figure 2A,C) in the calvarias. It is known that macrophages play a critical role in the inflammatory response and can be polarized into M1 and M2 types depending on the local microenvironment and that M1 macrophages secrete pro‐inflammatory cytokines such as TNF‐α, IL‐1β, IL‐6 and iNOS, while M2 macrophages produce anti‐inflammatory cytokines such as IL‐10 and IL‐4. We therefore investigated the effect of CA on macrophage polarization in vivo. qRT‐PCR results showed that *Tnf‐α*, *Il‐1β*, *Il‐6* and *Inos* (Figure 2D) were significantly increased, while *Il‐10* and *Arg‐1* (Figure 2E) were mildly decreased in the calvarial tissues of the model mice. After CA treatment, *Tnf‐α*, *Il‐1β*, *Il‐6* and *Inos* (Figure 2D) were significantly reduced, while *Il‐10* and *Arg‐1* (Figure 2D) were increased. In addition, ELISA analysis revealed that CA dose‐dependently attenuated the upregulation of IL‐1β, TNF‐α and IL‐6 (Figure 2F), and rescued the downregulation of IL‐10 and IL‐4 (Figure 2G) in the serum of the model mice. These results suggested that CA treatment may shift macrophage polarization from M1 to M2 in the Ti‐induced calvarial osteolysis in mice, thereby attenuating inflammation in PPO.

### 3.3 CA rescues the Ti particle‐induced bone formation deficiency in vivo

**FIGURE 3:**
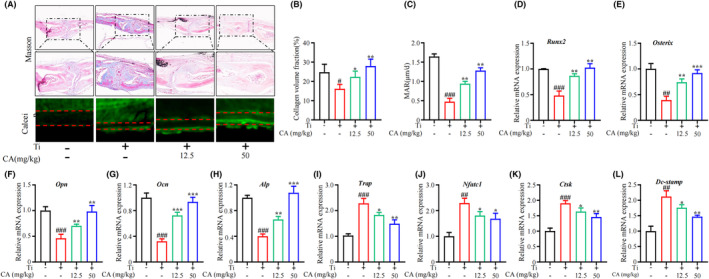
Effect of CA on Ti‐particle‐induced osteogenic deficits in murine calvarial tissue. (A) Representative images of Masson and Calcein staining of calvaria tissue. (B‐C) Quantitative analysis of collagen volume fraction (%) and MAR (μm/d). (D‐H) mRNA level of *Runx2*, *Osterix*, *Opn*, *Ocn* and *Alp* in mice calvaria tissue. (I‐L) mRNA level of *Trap*, *Nfatc1*, *Ctsk* and *Dc‐stamp* in mice calvaria tissue; *n* = 3 per group. ^#^ *p* < 0.05, ^##^ *p* < 0.01 versus Ctrl group. *p < 0.05, **p < 0.01 versus Ti group.

To investigate whether CA treatment could attenuate the osteogenesis ability in Ti‐particle‐induced osteolysis, we performed Masson staining and calcein staining to evaluate the osteogenic effect of CA on the Ti particle‐induced calvarial osteolysis in the mouse model. Masson staining showed that CA treatment rescued the downregulation of collagen fibre and collagen volume fraction (CVF) in Ti‐stimulated mice (Figure 3A,B). Fluorescent signals from calcein indicated that CA treatment restored the decreased distance between the two fluorescent signals in the calvarias tissue of the model mice (Figure 3A,C). In addition, CA treatment significantly alleviated mRNA levels of osteogenic‐related genes (*Runx2*, *Osterix*, *Opn*, *Ocn* and *Alp*) in the calvarias tissues of the model mice (Figure 3D–H). Moreover, CA treatment slightly alleviated the upregulated mRNA levels of osteoclast‐related genes (*Trap*, *Nfatc1*, *Ctsk* and *Dc‐stamp*) in the calvarias tissues of the model mice (Figure 3I–L). These results indicate that CA ameliorated decreased bone formation and slightly alleviated bone resorption in Ti‐treated mice in vivo.

### 3.4 CA attenuates Ti‐stimulated macrophage polarization from M2 to M1 in vitro

**FIGURE 4:**
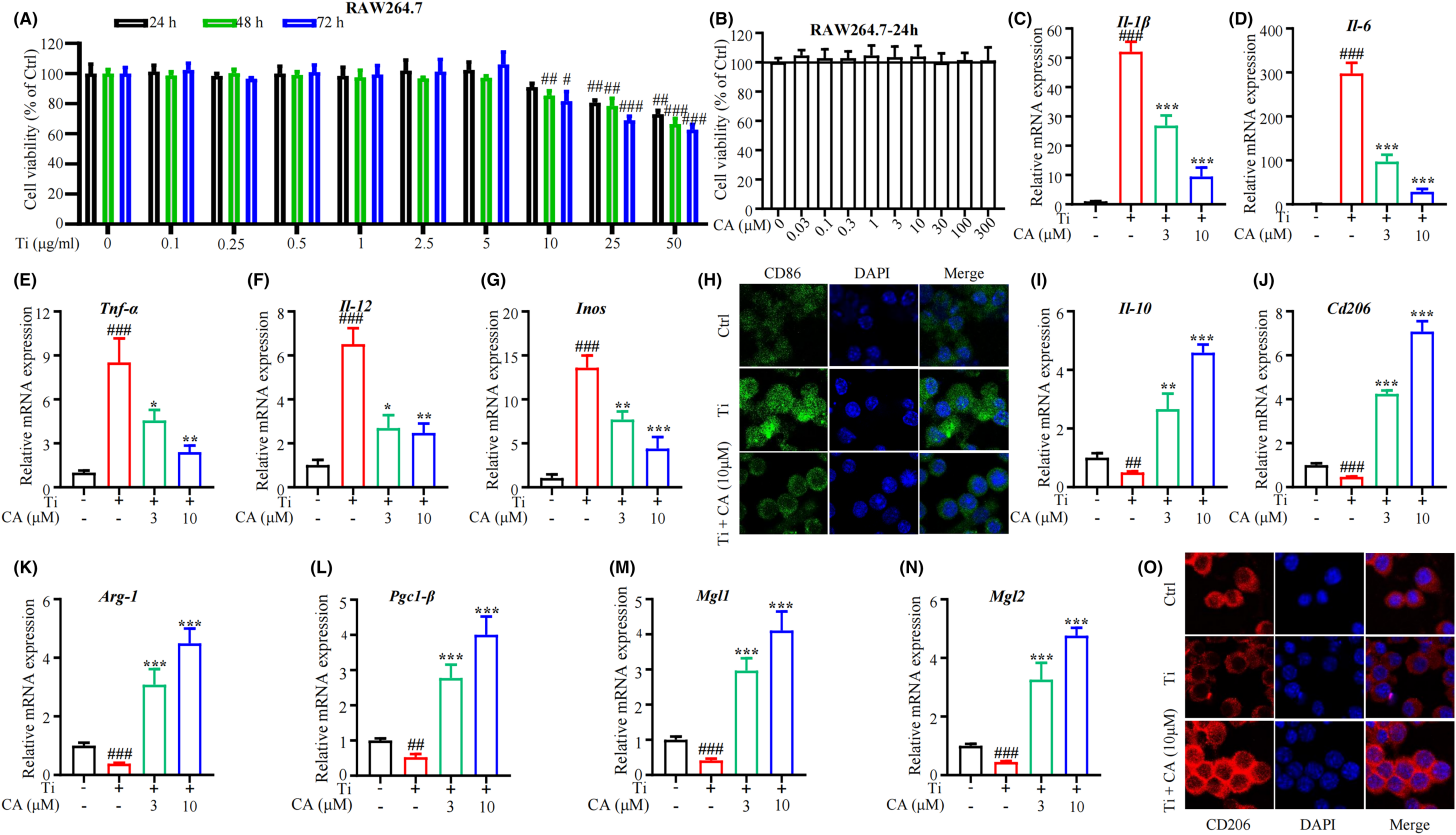
Effect of CA on Ti‐particle‐induced RAW264.7 cell polarization. (A) Cell viability of Ti particles on RAW264.7 cell. (B) Cell viability of CA on RAW264.7 cells. (C‐G) mRNA level of *Il‐1β*, *Il‐6*, *Tnf‐α*, *Il‐12* and *Inos* in RAW264.7 cells. (H) Immunofluorescence staining of CD86 (green) macrophages. (I–N) mRNA level of *Il‐10*, *Cd206*, *Arg‐1*, *Pgc1‐β*, *Mgl1* and *Mgl2* in RAW264.7 cells. (O) Immunofluorescence staining of CD206 (red) macrophages. *n* = 3 per group. ^#^ *p* < 0.05, ^##^ *p* < 0.01 versus Ctrl group. *p < 0.05, **p < 0.01 versus Ti group.

To determine an optimal concentration for Ti treatment in vitro, a range of concentrations (0.1, 0.25, 0.5, 1, 2.5, 5, 10, 25 and 50 μg/mL) of Ti were selected for the cell viability assay. The results showed that Ti treatment did not have significant cytotoxicity to RAW264.7 for up to 72‐h cell culture at concentrations below 10 μg/mL (Figure 4A). Therefore, the subsequent cell experiments were conducted using 5 μg/mL of Ti. The effect of CA on the viability of RAW264.7 macrophages was also assessed through CCK‐8 assay, and the results showed no significant cytotoxicity below the concentrations of 300 mM (Figure 4B). Previous research has demonstrated RAW264.7 cell can polarization from M2 to M1 upon Ti particle stimulus,^20^ we therefore incubated RAW264.7 cells with different concentrations of CA for 2 h, followed by stimulation with Ti particles for 30 min. The result of the qRT‐PCR assay showed a consistent result with previous studies that treatment with Ti alone increased M1 polarization‐associated genes (Figure 4C–G), and slightly inhibited M2 polarization‐associated genes (Figure 4I–N). In addition, CA treatment attenuated the upregulation of M1‐related genes (*Il‐1β*, *Il‐6*, *Tnf‐a*, *Il‐12* and *Inos*) in a dose‐dependent manner (Figure 4C–G). Moreover, M2‐related genes (*Il‐10*, *Cd206*, *Arg‐1*, *Pgc1‐β*, *Mgl1* and *Mgl2*) were increased after being treated with CA (Figure 4I–N). Immunofluorescence staining of macrophages M1 (CD86) and M2 (CD206) markers showed a similar result with qRT‐PCR (Figure 4H,O). These data suggest that CA converts macrophages from M1 to M2 in Ti‐stimulated RAW264.7 cells, which is in line with the above‐mentioned histological study.

### 3.5 Evaluation of osteogenic differentiation capacity in conditioned cultures

**FIGURE 5:**
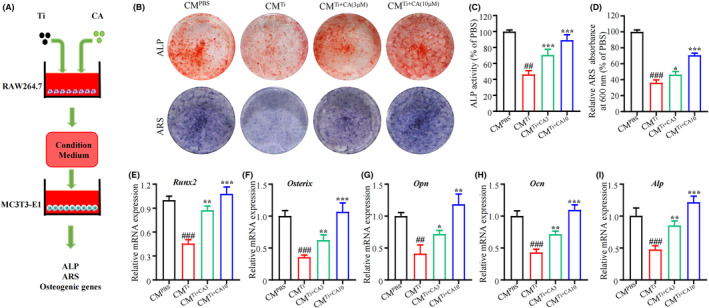
Effect of condition medium from CA and Ti‐particle‐stimulated RAW264.7 cells on osteogenic differentiation. (A) Schematic depiction of the experiment. (B) Representative images of ALP and ARS staining. (C) Quantitative analysis of ALP activity. (D) Quantitative analysis of ARS Absorbance at 600 nm. (E‐I) mRNA level of *Runx2*, *Osterix*, *Opn*, *Ocn* and *Alp* in MC3T3‐E1 cells. *n* = 6 per group. ^#^ *p* < 0.05, ^##^ *p* < 0.01 versus Ctrl group. *p < 0.05, **p < 0.01 versus Ti group.

Previous studies have reported the impact of macrophage polarization on osteogenic differentiation and subsequent bone regeneration.^21^, ^22^ We used the RAW264.7 cell culture supernatant as a CM to assess the immunomodulatory effect of CA on osteogenic differentiation in MC3T3‐E1 cells (Figure 5A). ALP and ARS staining was performed on the cultured cells in CM (Figure 5B). The ALP activity in the CM^Ti^ group was reduced compared to the CM^PBS^ group (Figure 5B,C). However, ALP activity was significantly increased in the CM^Ti+CA3^ group, followed by the CM^Ti+CA10^ group (Figure 5B,C). Furthermore, ARS staining revealed a decreased extracellular matrix mineralization (ECM) in the CM^Ti^ group, with a high level of calcium mineralization observed in the CM^Ti+CA3^ group and CM^Ti+CA10^ group (Figure 5B,D). qRT‐PCR was conducted to evaluate the impact of osteogenic transcription factors. Consistent with the result of ALP and ARS staining, mRNA levels of osteogenic genes (*Runx2*, *Osteorix*, *Opn*, *Ocn* and *Alp*) were reduced in the CM^Ti^ group (Figure 5E,I). However, a higher mRNA level of these genes was observed in the CM^Ti+CA3^ group, followed by the CM^Ti+CA10^ group (Figure 5E,I).

### 3.6 CA inhibits Ti‐particle‐induced NF‐κB signalling pathway and macrophage inflammatory response

**FIGURE 6:**
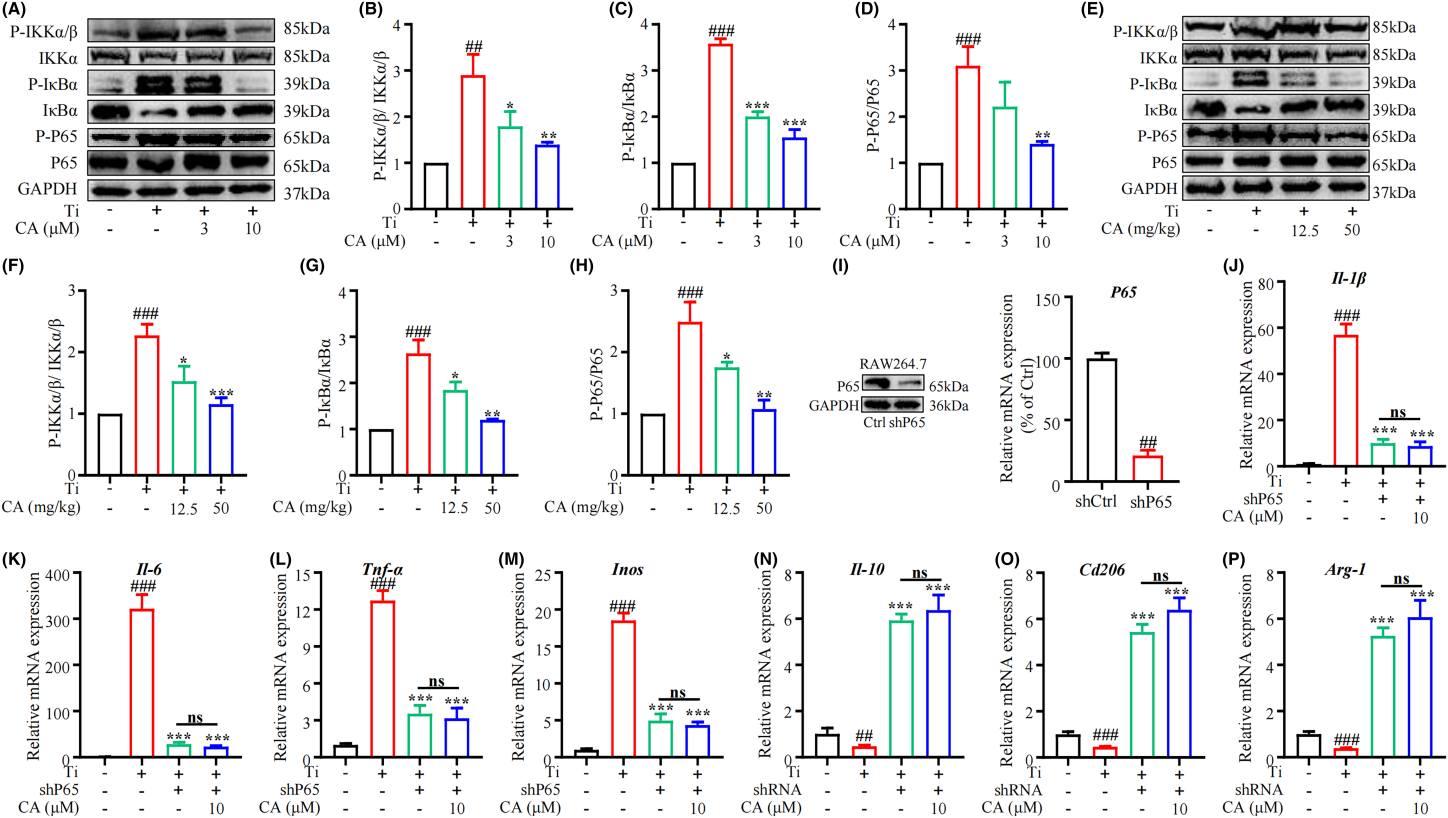
Effect of CA on Ti particles‐stimulated NF‐κB signalling pathway and inflammatory response. (A–D) Protein levels of P‐IKKα/β, IKKα, P65, P‐P65, IκBα and P‐IκBα stimulated by Ti with different concentrations of CA. (E–H) Protein levels of P‐IKKα/β, IKKα, IκBα, P‐IκBα, P65 and P‐P65 in the calvaria tissue of mice. (I) Protein and mRNA levels of P65 in the RAW264.7 cells. mRNA level of *Il‐1β* (J), *Il‐6* (K), *Tnf‐α* (L), *Inos* (M), *Il‐10* (N), *Cd206* (O) and *Arg‐1* (P) in RAW264.7 cells. *n* = 3, ^#^ *p* < 0.05, ^##^ *p* < 0.01 versus Ctrl group. *p < 0.05, **p < 0.01 versus Ti group.

The NF‐κB signalling pathway plays a crucial role in maintaining macrophage polarization homeostasis.^23^, ^24^ Ti particles have been shown to promote M1 macrophage polarization and inhibit M2 macrophage polarization via activation of the NF‐κB signalling pathway.^25^ Here, we evaluated the impact of CA on the NF‐κB signalling pathways stimulated by Ti particles. RAW264.7 cells were treated with or without CA for 2 h and then stimulated with Ti particles for 30 min. Subsequently, we detected the expression of NF‐κB signalling‐related proteins such as IKKα, P‐IKKα/β, IκBα, P‐IκBα, P65 and P‐P65. The result showed that the P‐IKKα/β, P‐P65 and P‐IκBα levels were elevated along with the decrease of IκBα levels in Ti‐stimulated cells (Figure 6A–D). However, we observed a concentration‐dependent mitigation of these alterations following CA treatment (Figure 6A–D). In addition, we detected NF‐κB signalling proteins in vivo, and found that CA relieved the activation of the NF‐κB signalling pathway in the calvarias tissues of model mice (Figure 6E–H). These results indicate that CA alleviated the activation of the NF‐κB signalling pathway by Ti particles, both in vitro and in vivo. To investigate whether CA regulated the homeostasis of M1/M2 macrophages through NF‐κB signalling, shRNA for P65 was developed to block this signalling pathway. RAW264.7 cells were transfected with control (shCtrl) or P65 (shP65) shRNA. Both western blot and qRT‐PCR analyses showed that P65 expression was significantly reduced in the knockdown group (Figure 6I). qRT‐PCR analysis revealed that in P65‐silenced cells, CA could not further inhibit the expression of M1 phenotype markers (*Il‐1β*, *Il‐6*, *Tnf‐α* and *Inos*) induced by Ti in p65‐silenced cells (Figure 6J–M). Moreover, CA could not further elevate Ti‐induced M2 phenotype markers (*Il‐10*, *Cd206* and *Arg‐1*) expression in p65‐silenced RAW264.7 cells (Figure 6N–P). The results suggest that CA attenuated Ti‐stimulated macrophage polarization via the NF‐κB signalling pathway.

### 3.7 CA cannot promote osteogenesis of MC3T3‐E1 cells cultured with condition medium from P65‐silenced macrophages

**FIGURE 7:**
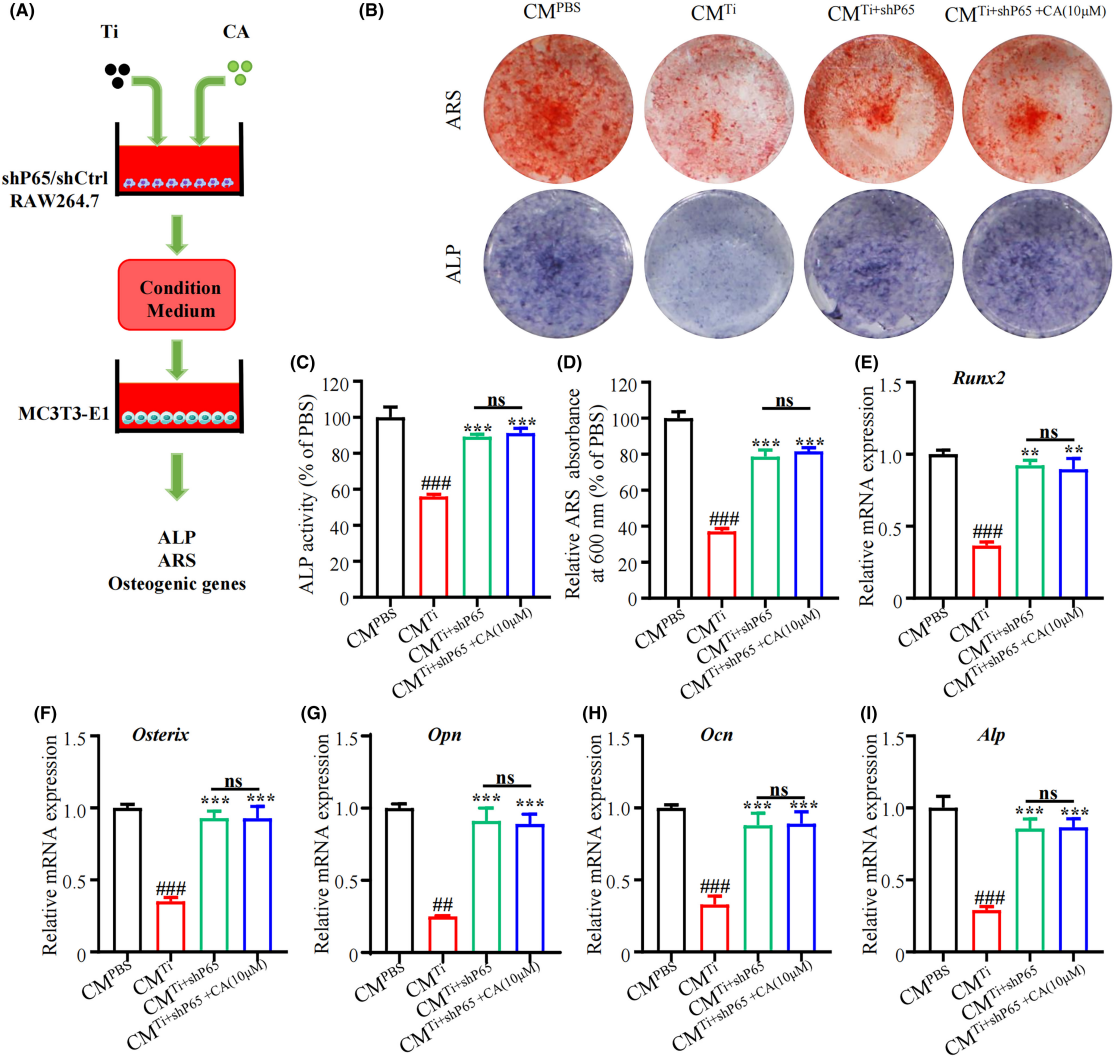
CA cannot promote osteogenesis in MC3T3‐E1 cells cultured with conditioned medium from P65‐silenced macrophages (A) Schematic depiction of the experiment. (B) Representative images of ALP and ARS staining. (C) Quantitative analysis of ALP activity. (D) Quantitative analysis of ARS absorbance at 600 nm. mRNA level of *Runx2* (E), *Osterix* (F), *Opn (*G), *Ocn (*H) and *Alp* (I) in MC3T3‐E1 cells. *n* = 3 per group. ^#^ *p* < 0.05, ^##^ *p* < 0.01 versus CM^PBS^ group. *p < 0.05, **p < 0.01 versus CM^Ti^ group.

To determine whether CM from Ti‐treated macrophage impaired osteogenesis through the NF‐κB signalling pathway, we stimulated control (shCtrl) or P65 (shP65) RAW264.7 cells with Ti and CA, and then collected the macrophage CM to stimulate MC3T3‐E1 cells (Figure 7A). The results of ALP and ARS staining showed that ALP activity and ECM in cultured MC3T3‐E1 cells in CM^Ti+shP65^ were elevated as compared with CM^Ti^‐treated MC3T3‐E1 cells (Figure 7B–D), while CM^Ti+CA+shP65^ could not further stimulate MC3T3‐E1 cells towards osteogenic differentiation compared to CM^Ti+shP65^ (Figure 7B–D). Consistent with the result of ALP and ARS staining, mRNA levels of osteogenic genes (*Runx2*, *Osteorix*, *Opn*, *Ocn* and *Alp*) showed a similar trend (Figure 7E–I). The results demonstrated that CA could not promote osteogenic differentiation of MC3T3‐E1 cells cultured with condition medium from P65‐silenced macrophages.

## 4 DISCUSSION

Osteolysis caused by wear debris is the leading cause of aseptic prosthesis loosening, which is a frequent reason for arthroplasty failure.^26^ Wear debris generated by the joint material can mediate inflammatory response and consequently induce osteolysis by stimulating multiple immune cells, among which macrophages play an essential role in osteolysis by phagocytosing wear particles to produce inflammatory factors that modulate the bone immune microenvironment.^27^ In this study, we evaluated the therapeutic effect of CA on Ti particle‐induced osteolysis, the immunoregulatory effect of CA on Ti particle‐induced inflammation and the subsequent effect on osteogenesis. It was found that CA could mechanistically reduce the upregulation of M1‐associated cytokines and slightly increase M2‐associated cytokines to alleviate bone deficits. In addition, the effect of CA on Ti particle‐induced macrophage polarization was found to be closely associated with the NF‐κB signalling pathway. These findings demonstrated that CA could alleviate osteogenic deficits by regulating macrophage polarization through the NF‐κB signalling pathway, thereby prolonging the life of the prosthesis.

CA is a representative phytoestrogen derived from *Astragalus membranaceous (Fisch.) Bunge*. It possesses various biological properties, including anti‐inflammatory, anti‐osteoporotic and antioxidant activities,^10^ suggesting that CA may have a potential anti‐osteolysis effect. Ti particles provide typical wear debris in arthroplasty, while the Ti particle‐induced mouse calvarial osteolysis model is a commonly utilized animal model to mimic PPO.^3^ Our experiment demonstrated the protective effect of CA in the Ti‐particle‐induced calvarial osteolysis mouse model, as evidenced by radiological examination and histomorphological analysis. In addition, we observed no significant adverse effects of CA on body weight and gross organ morphology (data not shown). Our experiment showed that CA was safe at the dose of exerting an anti‐PPO effect in the mouse model.

Depending on the stimulus, macrophages can polarize into pro‐inflammatory M1 or anti‐inflammatory M2.^28^ It has been reported that wear particles stimulate the differentiation of macrophages towards the M1 phenotype to express CD86 and CD80 and secrete various pro‐inflammatory cytokines such as IL‐1β, IL‐6, TNF‐α and iNOS. Whereas wear particles inhibit macrophage polarization towards the M2 phenotype to express CD206 and Arg‐1, and secrete IL‐4/IL‐10 and other anti‐inflammatory cytokines. The imbalance between M1 and M2 phenotypes and the subsequent secretion of inflammatory factors may impede osteogenesis and promote the progression of PPO.^29^ CA has been shown to inhibit the production of pro‐inflammatory cytokines in LPS‐stimulated macrophages.^30^, ^31^ However, the mechanism of CA action in attenuating osteolysis in PPO mice remains unclear. Our in vivo experiments showed that CA effectively inhibited the upregulation of M1‐associated cytokines and increased the downregulation of M2‐associated cytokines in calvarias tissue and sera of the model mice. Our in vitro experiments showed that CA effectively inhibited Ti‐stimulated M1‐associated cytokines and slightly increased M2‐associated cytokines in RAW264.7 cells. These results suggest that CA may exert its anti‐PPO effect by rebalancing the M1/M2 macrophage polarization, especially by reducing macrophage polarization towards M1.

Osteoblasts originating from mesenchymal stem cells play a crucial role in bone formation and an essential role in prosthetic stability.^32^ Macrophages play a central regulatory role in all stages of bone regeneration, and cytokines are involved in their action.^33^ Knowing that wear particle‐stimulated macrophage inflammation inhibits osteogenesis and regulates aseptic loosening,^34^ we, therefore, investigated the effect of macrophage‐derived cytokines on the osteogenic process using macrophage culture supernatants to create a CM. The result demonstrated that CA‐treatment rescued the downregulation of osteogenesis in the Ti‐induced osteolysis mouse model, as evidenced by qRT‐PCR and tissue staining analysis. In addition, our in vitro experiments revealed that CA + Ti‐induced macrophages were able to reverse the inhibitory effects of Ti‐induced macrophage supernatant on MC3T3‐E1 osteogenesis, as indicated by ALP staining, alizarin red staining and qRT‐PCR analysis. These results align with the finding of previous studies that macrophage polarization plays an immunomodulatory role in osteogenesis, which could be a possible mechanism for bone regeneration.^35^ Above all, our findings suggest that CA may alleviate osteolysis by regulating macrophage polarization homeostasis and, subsequently, osteogenic differentiation.

Osteoclasts are derived from myeloid progenitor cells, and the plasma membrane of osteoclasts displays V‐ATPase along the crease, which plays a crucial role in the lacunar acidification and phagocytosis process. An acidic environment aids in the dissolution of acidic plaque and stimulates cathepsin K to digest collagen, thereby promoting bone resorption.^36^ Previous research has established that CA suppresses the RANKL‐stimulated osteoclastogenesis in primary bone marrow macrophages.^37^ Our study found that CA treatment slightly reduced elevated osteoclast‐related genes in the calvaria model mice, suggesting its potential to inhibit osteoclast activity, which provides partial support for the suppressive role of CA in osteolysis. Further investigation into the interference with the V‐ATPase function is necessary.

Previous studies have indicated that the PPO microenvironment induced by wear particles stimulates macrophages to activate the downstream NF‐κB pathway, ultimately triggering the transcription of target genes. This promotes M1 macrophage polarization and inhibits M2 polarization, leading to an increase in multiple pro‐inflammatory cytokines and a decrease in anti‐inflammatory cytokines.^38^ It was found in our study that CA inhibited the Ti‐stimulated imbalance of macrophage polarization and NF‐κB signalling pathway activation. Similar inhibitory effects have been observed in other diseases. For instance, CA ameliorated the inflammatory response in diabetic kidney injury by inhibiting the NF‐κB signalling pathway.^39^ To investigate whether CA regulates macrophage polarization through other signalling pathways, we used shRNA to interfere with the macrophage P65 expression, and found that CA could not further inhibit the Ti‐stimulated polarization of P65‐silenced macrophages towards M1 and promote cell polarization towards M2. Further investigation into the effect of these macrophage supernatants on osteogenesis revealed that P65‐silenced macrophage supernatants could not further promote the osteogenesis of MC3T3‐E1 cells. These results suggest that CA alleviated PPO by regulating macrophage polarization homeostasis through inhibiting NF‐κB/P65 transcriptional activation, thereby reducing inflammation and inhibiting insufficient bone formation.

In this study, we demonstrated the therapeutic effects of CA and possible molecular mechanisms in an experimental mouse model of PPO, which may provide a new option for PPO treatment. However, there are some limitations in our studies. We investigated the effect of CA on macrophages and the regulation of osteogenesis through macrophage polarization. Although multiple cell types are involved in regulating the microenvironment of osteolysis, such as osteoclasts, B cells and T cells, further investigation is required to determine the regulatory effect of CA on these cells. In addition, the mouse model of calvarias osteolysis used represents an acute inflammatory condition rather than a chronic inflammatory condition with aseptic loosening. Thus, a more appropriate model is required to evaluate the long‐term efficacy of CA in future studies.

In conclusion, this study explored the therapeutic effect of CA in an experimental mouse model of PPO and found that CA could mechanically alleviate osteogenic deficit in PPO by regulating macrophage polarization homeostasis through the NF‐κB signalling pathway. These findings may provide a therapeutic agent candidate for PPO.

## AUTHOR CONTRIBUTIONS

**Hui Jiang:** Formal analysis (equal); funding acquisition (equal); project administration (equal); writing – original draft (equal); writing – review and editing (equal). **Yang Wang:** Data curation (equal); investigation (equal); validation (equal). **Zhao Tang:** Data curation (equal); funding acquisition (equal); software (equal); visualization (equal). **Xianjiang Peng:** Data curation (equal); investigation (equal); software (equal). **Chan Li:** Methodology (lead). **Yangjie Dang:** Validation (equal); writing – review and editing (equal). **Rui Ma:** Conceptualization (lead); data curation (lead); resources (lead); supervision (lead).

## FUNDING INFORMATION

This work was supported by the Research Fund from Jinling School of Clinical Medicine (No. 22LCZLXJS44 to Hui Jiang), the Special Fund for Prevention and Treatment of Military Training Injury (No. 20XLS18 to Hui Jiang) and the Research Fund from Jinling School of Clinical Medicine (No. 22LCZLXJS31 to Zhao Tang).

## CONFLICT OF INTEREST STATEMENT

The authors hereby declare no conflict of interest.

## Supporting information


Table S1.

## Data Availability

The data generated and/or analysed during this study are available from the corresponding author on reasonable request.
