# Supplementary material for: Calycosin alleviates titanium particle‐induced osteolysis by modulating macrophage polarization and subsequent osteogenic differentiation
Source: J Cell Mol Med. 2024 Mar 17;28(7):e18157. doi: 10.1111/jcmm.18157 (PMC10945085; doi:10.1111/jcmm.18157)
Supplement: Supplementary file 1 — Table S1. [file JCMM-28-e18157-s001.docx]

Supplement Table1. Primers used for qRT-PCR analysis

| Oligo sets mouse | Forward primer (5’ to 3’) | Reverse primer (5’ to 3’) |
| --- | --- | --- |
| *Il-1β* | ATGAAGGGCTGCTTCCAAAC | TCTCCACAGCCACAATGAGT |
| *Il-6* | GGAGCCCACCAAGAACGATA | ACCAGCATCAGTCCCAAGAA |
| *Tnf-α* | CTCATGCACCACCATCAAGG | ACCTGACCACTCTCCCTTTG |
| *Inos* | GCGCTCTAGTGAAGCAAAGC | TGCAACAGCTGAGGAAGGACTTGA |
| *Il-12* | GTGGAATGGCGTCTCTGTCT | GGTCTGGTTTGATGATGTCCCT |
| *Arg-1* | TGGCTTGCGAGACGTAGAC | GCTCAGGTGAATCGGCCTTT |
| *Il-10* | CTGGACAACATACTGCTAACCG | GGGCATCACTTCTACCAGGTAA |
| *Cd206* | CAGCGGTTGGCAGTGGA | CAGCTGATGGACTTCCTGGTAAG |
| *Pgc1β* | CTTCCGTTGGCCCAGATAC | CTGCTGGGCCTCTTTCAGTA |
| *Mgl1* | TGCAACAGCTGAGGAAGGACTTGA | AACCAATAGCAGCTGCCTTCATGC |
| *Mgl2* | GCATGAAGGCAGCTGCTATTGGTT | TAGGCCCATCCAGCTAAGCACATT |
| *P65* | ACAGACCCAGGAGTGTTCACAGA | CATGGACACACCCTGGTTCAG |
| *Opn* | GAGGGCTTGGTTGTCAGC | CAATTCTCATGGTAGTGAGTTTTCC |
| *Alp* | CCAACTCTTTTGTGCCAGAGA | GGCTACATTGGTGTTGAGCTTTT |
| *Runx2* | TCGGAGAGGTACCAGATGGG | TGAAACTCTTGCCTCGTCCG |
| *Ocn* | CTGACCTCACAGATCCCAAGC | TGGTCTGATAGCTCGTCACAAG |
| *Osterix* | GGAGGTTTCACTCCATTCCA | TAGAAGGAGCAGGGGACAGA |
| *Dc‑stamp* | CTTCCGTGGGCCAGAAGTT | AGGCCAGTGCTGACTAGGATGA |
| *Nfatc1* | CCGTTGCTTCCAGAAAATAACA | TGTGGGATGTGAACTCGGAA |
| *Ctsk* | GGCTGTGGAGGCGGCTAT | AGAGTCAATGCCTCCGTTCTG |
| *Trap* | AGGGTGTGATGGTGGGAATG | GCTGGGGTGTTGAAGGTCTC |
| *β-actin* | GGCTGTATTCCCCTCCATCG | CCAGTTGGTAACAATGCCATGT |
